# The Efficacy of 532/755 nm Laser Therapy for Facial Pigmented and Vascular Lesions: A Systematic Review and Meta-Analysis

**DOI:** 10.3390/jcm14082546

**Published:** 2025-04-08

**Authors:** Piotr Zawodny, Paweł Zawodny, Monika Kulaszyńska, Elżbieta Stój, Anna Knap-Czechowska, Karolina Skonieczna-Żydecka, Jerzy Sieńko

**Affiliations:** 1 Medical Center Zawodny Clinic, Ku Słońcu 58, 71-047 Szczecin, Poland; estetic@estetic.pl (P.Z.);; 2 Department of Plastic, Endocrine and General Surgery, Pomeranian Medical University in Szczecin, Siedlecka 2, 72-010 Police, Poland; 3 Department of Biochemical Science, Pomeranian Medical University in Szczecin, Broniewskiego 24, 71-460 Szczecin, Poland; 4 Institute of Physical Culture Sciences, University of Szczecin, 70-453 Szczecin, Poland

**Keywords:** aesthetic medicine, laser therapy, 532 nm, 755 nm, pigmented lesions, vascular lesions, meta-analysis

## Abstract

**Objectives**: The increasing demand for aesthetic medical treatments has led to advancements in laser therapies, particularly for pigmented and vascular facial lesions. This study compares the efficacy of 532 nm and 755 nm lasers for treating facial pigmented and vascular lesions. **Methods**: Data extraction was performed in accordance with the Preferred Reporting Items for Systematic Reviews and Meta-Analyses (PRISMA). Primary outcomes included the (i) improvement and (ii) pain sensation. Secondary outcomes included the occurrence of adverse events. A random-effects meta-analysis was conducted for outcomes where two or more studies provided data. Continuous outcomes were analyzed using pooled standardized mean differences, while nominal outcomes were assessed using pooled risk ratios in endpoints, using observed cases only. **Results**: A total of 16 studies involving 509 participants were included. No significant difference in overall improvement between the 532 nm and 755 nm lasers was found. Subgroup analysis showed a slight advantage in the 755 nm group for lesion improvement (RR = 1.512, 95% CI [1.070, 2.136], *p* = 0.019). Adverse event occurrence was minimal, with no significant differences between the laser types, however the pain score was higher for the 532 nm laser (SMD = −1.336, SE = 0.636, 95% CI [−2.582, −0.09], *p* = 0.036). No publication bias with respect to any evaluated intervention was detected. **Conclusions**: This meta-analysis concludes that both 532 nm and 755 nm lasers are effective in treating facial pigmented and vascular lesions, with the 755 nm laser showing a slight advantage and the 532 nm one producing more painful experiences but fewer adverse events in the case of vascular lesions.

## 1. Introduction

Aesthetic medicine is a rapidly growing field, with an increasing number of patients undergoing treatments to enhance their appearance each year [1]. Aesthetic medicine contributes to this holistic view of health, addressing not only the physical aspects but also the mental and social dimensions, as physical appearance impacts many aspects of an individual’s life. Pigmented and vascular skin lesions are common signs of aging that can negatively affect self-esteem and psychological well-being. With the advent of laser therapy, these lesions can be treated, reducing the impact on patients’ mental health and self-image [2].

Pigmented and vascular lesions are primarily aesthetic concerns, and their incidence increases with age. Hyperpigmentation can result from various factors, including physical or chemical agents, medications, hormonal imbalances, inflammation, metabolic disorders, and vitamin deficiencies [3]. Vascular abnormalities, such as telangiectasias, arise from increased venous pressure, incompetent veins, or arteriovenous fistulas, and may coexist with genetic conditions or be triggered by systemic diseases or medication side effects [4].

Laser treatment has become an established method in dermatology, utilizing specific wavelengths to deliver energy to targeted tissues. The effect of the laser depends on the interaction of the laser’s energy with the tissue, which can result in various outcomes, such as photothermal (heat), photomechanical (shock waves), photochemical (molecular bond disruption), or photobiomodulation (cell stimulation) [5]. The choice of wavelength, pulse duration, and energy determines the treatment’s success by targeting and destroying specific tissues while minimizing damage to surrounding areas [6]. Picosecond lasers, for example, have a shorter pulse width and higher peak power density compared to nanosecond lasers, providing a greater photomechanical effect and less heat diffusion to surrounding structures [7]. Previous studies have demonstrated that lasers can selectively target melanin in pigmented lesions and hemoglobin in vascular lesions, leading to the destruction of unwanted tissues through photocoagulation and selective photothermolysis [8,9].

While it is indeed clinically intuitive and already known that lesion type/depth and skin type guide laser selection, to our knowledge, no prior meta-analysis has systematically synthesized comparative data on the efficacy, safety, and pain levels of 532 nm and 755 nm lasers specifically for facial pigmented and vascular lesions. Thus, this meta-analysis aims to compare the efficacy of two commonly used lasers—532 nm and 755 nm—in the treatment of pigmented and vascular lesions on the face. This analysis seeks to systematize the findings from prior research to determine which laser wavelength offers superior outcomes in treating facial pigmented and vascular lesions.

## 2. Materials and Methods

### 2.1. Search Strategy and Inclusion Criteria

Two independent authors (KSZ, PZ) conducted a systematic search of PubMed, Embase, and CINAHL databases from inception until 1 November 2023 for randomized controlled trials (RCTs) focusing on the use of 532/755 nm laser therapy in the treatment of vascular and pigmented lesions on the face across all genders. The lesions which were of the interest included the following:

- hemangiomas
- vascular malformations
- hyperpigmentation
- freckles
- chloasma/ostuda/melasma
- nevoid hypermelanosis
- ephilides
- lentigines
- port wine stain
- nevus of Ota-like macules

The specific search terms used in PubMed and CINAHL were as follows: ( vascular lesion OR lesion, vascular OR skin lesion, vascular OR vascular lesion OR vascular skin lesion OR pigmented lesion OR telangiectasia OR telangiectasia OR telangiectasis OR teleangiectasia OR teleangiectasis OR hemangioma OR benign hemangioma OR haemangioma OR haemangioma planum OR hemangioma OR hemangioma planum OR hemangiomata OR hemangiomatous OR ossifying haemangioma OR ossifying hemangioma OR hyperpigmentation OR hyper pigmentation OR hyperpigmentation OR skin hyperpigmentation OR chloasma OR chloasma OR mask of pregnancy OR melasma OR ephelis OR ephelides OR ephelis OR freckle OR freckles OR skin freckling) AND (face OR face OR face form OR face shape OR facial form OR facial shape OR human face OR midface OR midfacies) AND ((laser OR beam, laser OR laser OR laser adapter OR laser adaptor OR laser associate OR laser beam OR laser device OR laser holder OR laser irradiation OR laser radiation OR laser ray OR lasers OR radiation, laser) AND 532 nm OR 755 nm). In Embase, similar terms were used but adjusted according to the Embase indexing system. The search was limited to human studies.

The search was supplemented by manual screening of relevant systematic reviews and meta-analyses. Zotero reference manager (version 6.0.36) was used for deduplication of results.

The inclusion criteria were as follows: (i) full-text, randomized, controlled trials involving the use of 532/755 nm laser therapy on facial lesions; (ii) studies comparing laser therapy against other treatments, i.e., different laser settings; (iii) studies providing efficacy and/or safety data; and (iv) publications in English. Studies were excluded if they were review articles, commentaries, editorials, case reports with fewer than 10 patients, or involved additional interventions that could affect facial skin lesions, other than laser therapy. The protocol of the present systematic review was neither registered in any of the databases nor previously published. However, along with the results, each step is available upon reasonable request.

### 2.2. Data Extraction and Outcomes

Data extraction was performed independently by two authors (KSZ, PZ) using a standardized data extraction form pre-prepared in an Excel file. The extracted data included study design, patient demographics, treatment protocols, and outcome measures related to efficacy and safety.

For the evaluation of risk of bias (ROB), the number of low ROB assessments was reported according to predefined criteria.

Primary outcomes included the improvement, described as any of kind (reduction in lesion size/intensity; improvement in skin appearance), and pain sensation. Secondary outcomes included the occurrence of adverse events and any other treatment-related outcome reported by the authors. Any discrepancies between the two reviewers (KSZ, PZ) during data extraction or risk of bias assessment were resolved through discussion. When consensus could not be reached, a third reviewer (JS) acted as an arbitrator. This step was performed in accordance with the Preferred Reporting Items for Systematic Reviews and Meta-Analyses (PRISMA). In the case of missing data, study authors were contacted via email, twice, two weeks apart.

### 2.3. Data Synthesis and Statistical Analyses

Data were synthesized using Comprehensive Meta-Analysis software (Biostat, Englewood, NJ, USA; version 4). A random-effects meta-analysis was conducted for outcomes where two or more studies provided data. For continuous variables, data were meta-analyzed only if means and standard deviations were provided. Descriptive data were presented in tabular form.

Continuous outcomes were analyzed using pooled standardized mean differences (SMD), while nominal outcomes were assessed using pooled risk ratios (RR) in endpoints, using observed cases (OC) only. Subgroup analysis for the 532/755 nm laser types where conducted where possible, i.e., for outcomes presented in studies of both types. For outcomes reported only in either 532 or 755 nm, no subgroup analyses was performed. To understand the relationship between effect sizes and various predictors, we fit random-effect meta-regression models where applicable. Meta-regression variables included pulse duration (ps vs. ns vs. ms) and lesion type (vascular vs. pigmented vs. mixed). These analyses were undertaken to inspect sources of heterogeneity.

The between-study variance (τ^2^) was estimated using the method of moments as described by DerSimonian and Laird [10]. The assumption of homogeneity of effects was evaluated using the Q statistic with k-1 degrees of freedom, where k represents the number of studies [11]. For nominal outcomes, the overall risk ratio (RR) was calculated. A two-tailed Z test was employed to assess the null hypothesis that the summary effect is zero. To determine if publication bias might have impacted the results, funnel plots were examined. Additionally, Egger’s regression test [12] and, if needed, Duval and Tweedie’s trim and fill method [13] were applied to further assess potential publication bias. In the sensitivity analysis, each study was sequentially excluded, and the effect size was recalculated to assess its impact on the overall effect size. All analyses were conducted as two-tailed tests with an alpha level set at 0.05.

### 2.4. Risk of Bias

The evaluation of risk of bias for each study was conducted using the Cochrane Risk-of-Bias Tool for randomized trials [14]. This tool assesses bias across several domains integral to randomized controlled trials, including selection bias, performance bias, detection bias, attrition bias, reporting bias, and other potential sources of bias. Information on randomization, allocation, blinding, attrition, and reporting practices was extracted from each study. Each domain was assigned a risk rating of “Low”, “High”, or “Unclear” based on the quality of the information provided in the study reports. “Low Risk” indicates that the study methodology effectively mitigated potential bias in that domain, “High Risk” suggests potential bias, and “Unclear” is used when information was insufficient for assessment.

## 3. Results

### 3.1. Search Results

**Figure 1:**
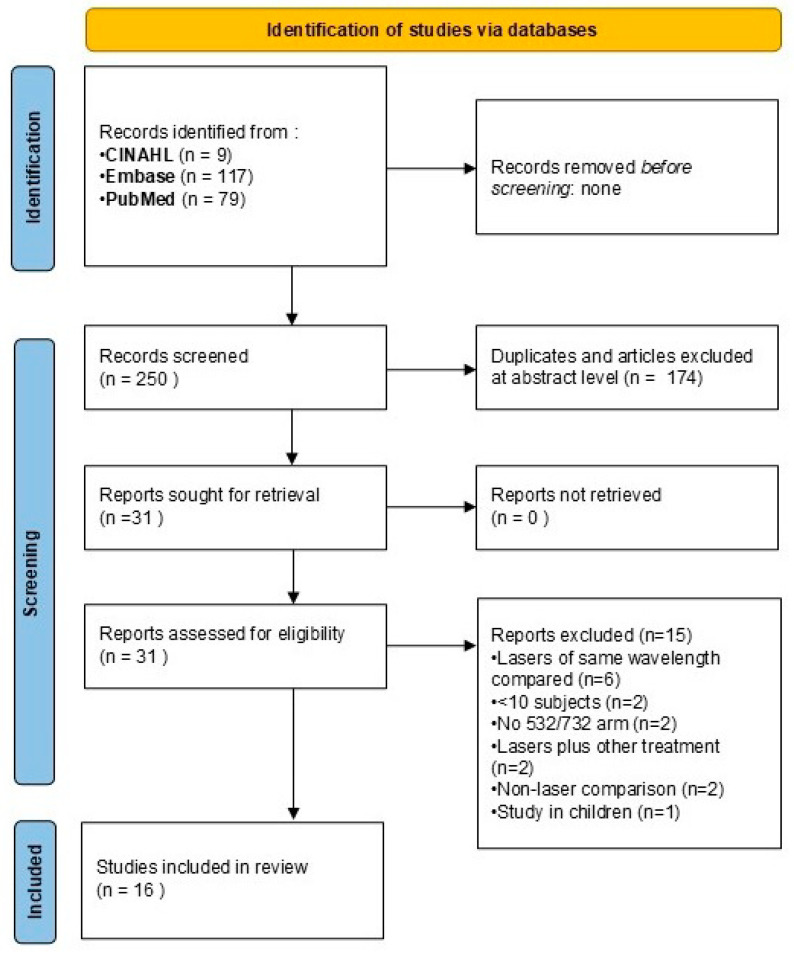
Study flow chart.

The process began with identifying potentially relevant articles from three databases: PubMed (79 hits), Embase (117 hits), and CINAHL (9 hits), totalling 205 articles. These articles were screened based on their abstracts and titles. From these initial articles, duplicates and those deemed irrelevant at the abstract level were excluded, reducing the number to 31 full-text articles that were retrieved for further evaluation of eligibility. During the eligibility assessment, these 31 articles were further scrutinized. None of the articles were identified via hand search. Of the 31 full-text articles assessed, 15 were excluded for various reasons: comparison of lasers of the same wavelength (6 articles), studies with fewer than 10 subjects (2 articles), absence of a 532/755 nm laser treatment arm (2 articles), lasers used in conjunction with other treatments (2 articles), non-laser comparisons (2 articles), and 1 study involving children. Finally, 16 articles were included in the meta-analysis (Figure 1).

### 3.2. Study and Subject Characteristics

Altogether, 16 studies (32 arms) were included in the present study. These included the total number of patients (n = 535) that were recruited and 509 who entered the final synthesis. Studies were conducted in the USA (n = 6), the United Kingdom (n = 3), China (n = 2), Korea (n = 2), Iraq (n = 1), Italy (n = 1), or multiple clinical centers (n = 1) and predominantly sponsored by academic funds (n = 9, 56.25%). There were patients of both sexes included, with the mean presence of males equal to around 12.0%. The highest mean age of the study participants was 57 years. The majority of studies were of split-face type (n = 12; 62.5%). In n = 4 studies, different patients were subjected to tested interventions. Overall, in n = 10 studies, the 532 nm laser was tested. The clinical efficacy of the 755 nm laser was tested in n = 6 studies. Data on other studies and participants’ characteristics are summarized in Table 1.

### 3.3. Clinical Efficacy of 532 and 755 Lasers Compared to Other Lasers

#### 3.3.1. Improvement

Across the studies, laser treatments showed varying degrees of efficacy. This depended on the type of skin lesions and the laser wavelength used, the latter one being the major aim of the present study. Also, the type of tool used to evaluate the efficacy influenced the results. Al-Dhalimi and Al-Janabi (2016) [15], who evaluated port wine stains, demonstrated that the 532 nm laser had a lower improvement score compared to the 1064 nm laser, measured via VAS, however a better (“excellent”) percentage improvement was noted for the 532 nm option (28.5% vs. 0%). Nevertheless, any degree of improvement was noted for all 532 nm treatments, and the outcomes were similar for 13 out of 14 cases. Bohnert et al. (2018) [16] showed better improvement with 532 nm-treatment of the side of face covered with solar lentigines as compared to a 1064 nm approach. In contrast, Felton et al. (2014) [18] reported that the 1064 nm wavelength achieved the highest improvement (97%) for nevus of Ota compared to shorter wavelengths, with rates of 90% and 75% for the 532 nm and 755 nm devices, respectively. Nam et al. (2019) [21] reported comparable improvements in erythema for the 532 nm KTP diode laser and the 595 nm pulsed dye laser (PDL) using both evaluation techniques, i.e., colorimeter analysis and photographic inspection performed by two independent dermatologists. Meanwhile, Uebelhoer et al. (2007) [23] pointed out that the KTP laser achieved 85% clearing for telangiectasia after three treatments compared to 75% for PDL. However, the authors provided no standard deviations with respect to these measurements and thus the results were not included in the synthesis here. West and Alster (1998) [24] observed the long-pulsed dye laser yielding a higher clearance (up to 95% expressed using a 4-point Likert scale) for facial and leg telangiectasias than the KTP laser. As the authors merged data here for low extremities and face, we could not form separate conclusions for facial lesions. As for the 755 nm laser, Bonan et al. (2021) [25] found the MoveoPL mode of the 755 nm alexandrite laser provided superior improvement for benign hyperpigmentation, as compared to the single-pass mode. Levin et al. (2016) [29] noted similar improvement scores for both the picosecond and Q-switched lasers in treating pigmentary disorders, each showing approximately 50% clearance. Ma et al. (2022) [22] found the 755 nm laser achieved higher mean improvement scores (3.78 on a 1–5 scale) compared to the 532 nm picosecond Nd: YAG laser (3.11 on a 1–5 scale) for freckles. Table 2 provides data on the improvement measurements with the name of the tools used.

##### Improvement Score

**Figure 2:**
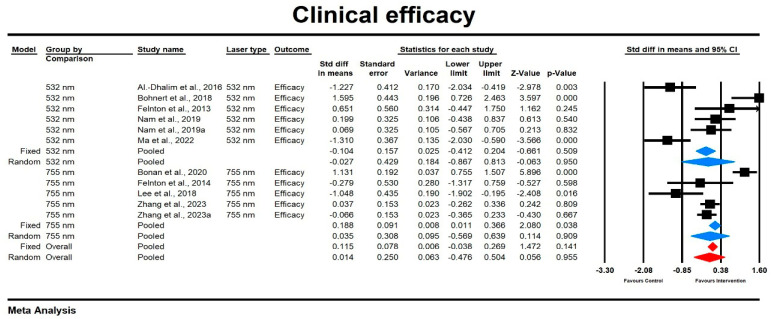
The effect size standardized mean differences (SDM) for the improvement score in patients subjected to the 532/755 nm (intervention) vs. the comparator (control) [15,16,18,21,22,25,27,30].

Using a mixed-effects model, we found that the overall effect size for the improvement score was low and did not vary significantly by laser type (SMD = 0.014, SE = 0.25, 95% CI [−0.476, 0.504]; *p* = 0.906). The heterogeneity among studies was high, with I^2^ = 86.7% and a significant Q-value (Q = 86,05, *p* = 0.000). Subgroup analysis indicated that the effect size was slightly higher in the case of the 755 nm laser compared to the 532 nm group, though both were not statistically significant (532 nm: SMD = −0.027, SE = 0.429, 95% CI [−0.867, 0.813], *p* = 0.895; 755 nm: SMD = 0.035, SE = 0.308, 95% CI [−0.569, 0.639], *p* = 0.909). The results are presented in Figure 2. The findings of the sensitivity analysis indicate that individual studies do not have a differential impact.

Funnel plot inspection revealed no significant publication bias, as indicated by Egger’s test (*p* = 0.55). The plot was symmetric, further suggesting minimal bias (see Supplemental Figure S1).

##### Relative Risk for Improvement

**Figure 3:**
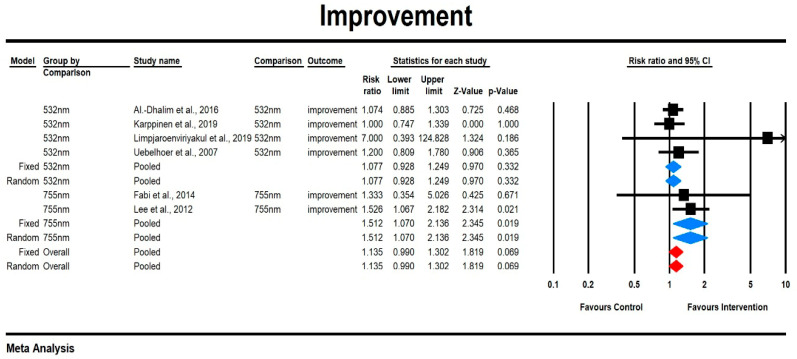
The effect size risk ratios (RR) for the improvement in patients subjected to 532/755 nm (intervention) vs. comparator (control). Examination of the funnel plot revealed no significant publication bias, as confirmed by Egger’s test (*p* = 0.17) (see Supplemental Figure S2) [15,19,20,23,26,28].

Using a mixed-effects model, we observed that the relative risk for the improvement was small and did not significantly differ between laser types, although there was a trend toward greater improvement with the 755 nm laser (RR = 1.135, 95% CI [0.99, 1.302], *p* = 0.069). The heterogeneity among studies was low, with I^2^ = 6.122% and a non-significant Q-value (Q = 5.326, *p* = 0.327). Subgroup analysis showed that the effect size was slightly higher for the 755 nm laser compared to the 532 nm laser, with the former showing statistical significance (755 nm: RR = 1.512, 95% CI [1.070, 2.136], *p* = 0.019; 532 nm: RR = 1.077, 95% CI [0.928, 1.249], *p* = 0.332). The results are presented in Figure 3. The sensitivity analysis findings suggest that individual studies do not exert a differential impact.

#### 3.3.2. Pain

Similarly to improvement indices, pain ratings also varied across studies depending on laser type. Butler et al. (2006) [17] found that patients rated the 532 nm laser as slightly more painful than IPL, with mean pain scores of 5.27 and 4.4 out of 10 Likert scale, respectively. Nevertheless, no standard deviations were reported; thus, these data were not meta-analyzed here. A group studied by Karppinen et al. (2019) [19] rated the 585 nm laser as significantly more painful compared to the KTP laser (532 nm), with scores of 67.7 vs. 34.6 in a 10-point Likert scale evaluation. In contrast, Ma et al. (2022) [22] found only a slight, non-significant difference in pain between QSAL (3.94) and PS532 (3.56), as measured on a numerical visual analog scale. Nam et al. (2019) [21] found that the KTP diode laser was rated less painful (1.49) than the 595 nm PDL (3.73) in treating telangiectatic erythema, similar to Uebelhoer et al. (2007) [23] who reported that more patients found the PDL slightly more painful than KTP for telangiectasia treatments. However, the latter result was not assessed with a pain score.

Bonan et al. (2021) [25] reported that the MoveoPL mode of the 755 nm alexandrite laser was less painful (1.8 on a 0–5 scale) compared to the single-pass mode (3.1 on a 0–5 scale). But Zhang et al. provided data that the 730 nm picosecond laser was less painful than the 755 nm approach (4.69 vs. 5.65, as evidenced with the VAS).

##### Pain Score

**Figure 4:**
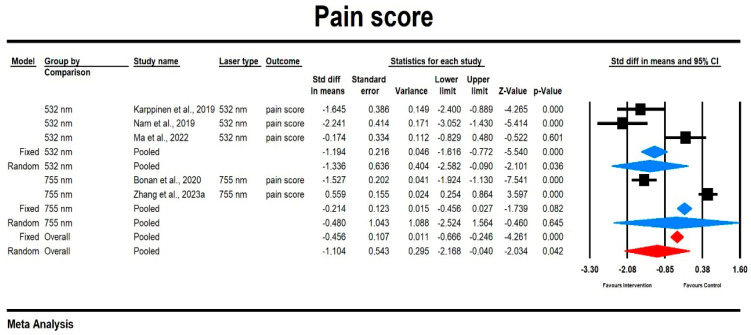
The effect size (SDM) for the pain score pain in patients subjected to 532/755 nm (intervention) vs. comparator (control) [19,21,22,25,30].

Using a mixed-effects model, we observed that the overall effect size for the pain score was small and did show significant differences between laser types (SMD = −1.104, SE = 0.543, 95% CI [−2.168, 0.064], *p* = 0.042). There was considerable heterogeneity among the studies, with I^2^ = 95.977% and a statistically significant Q-value (Q = 99.419, *p* = 0.000). Subgroup analysis revealed that the effect size was greater in the 532 nm group compared to the 755 nm group, but only the 532 nm result was statistically significant (532 nm: SMD = −1.336, SE = 0.636, 95% CI [−2.582, −0.09], *p* = 0.036; 755 nm: SMD = −0.480, SE = 1.043, 95% CI [−2.524, 1.564], *p* = 0.645). The results are displayed in Figure 4. The results of the sensitivity analysis indicate that individual studies do not have a significant influence on the overall effect.

Funnel plot inspection revealed no significant publication bias as indicated by Egger’s test (*p* = 0.248). The plot was symmetric, further suggesting minimal bias (see Supplemental Figure S3).

#### 3.3.3. Adverse Events

Safety profiles were generally favourable, though the authors indeed reported some more frequent adverse effects, but predominantly of a mild nature. Al-Dhalimi and Al-Janabi (2016) [15] reported no long-term adverse events, although one case of temporary hyperpigmentation was noted with the 532 nm laser (resolved with hydroquinone cream 4% and no scar). In Bohnert et al.’s [16] study, all patients experienced mild erythema and swelling irrespective of the laser used. Butler et al. [17] noted that erythema and edema, both in the infraorbital area of the KTP-treated side, occurred in two patients. In Karppinen et al.’s study (2019) [19], the PhotoLase yellow laser was associated with more blistering than the KTP laser (8 versus 3 participants), though erythema (17 vs. 16) and crusting (7 vs. 9) rates were similar for both lasers. In the present meta-analysis, data for only blistering were used for calculations.

Ma et al. (2022) [22] reported transient erythema and crusting with both lasers, with no long-term pigmentation changes or scarring. However, two cases developed transient hyperpigmentation on 532 nm-laser side. This was the case in only one man in the 755 nm-treated group. Uebelhoer et al. (2007) [23] noted more prolonged erythema (up to 5 days post treatment) and swelling on the KTP-treated side, though these effects resolved within days. In the present synthesis, data for erythema incidence were used.

In the case of the 755 nm approach, Bonan et al. [25] found a similar incidence of adverse events on the face (2% vs. 2% excluding erythema analyzed as score) between lasers used. Lee et al. [27] demonstrated that mild erythema and swelling without petechiae was present in all patients irrespective of the side of the face. Levin et al. (2016) [29] also reported only temporary side effects for both picosecond and Q-switched lasers, with no long-term complications recorded. However, dyspigmentation only for the Q-switched nanosecond treatment group was noted.

##### Relative Risk for Adverse Events

**Figure 5:**
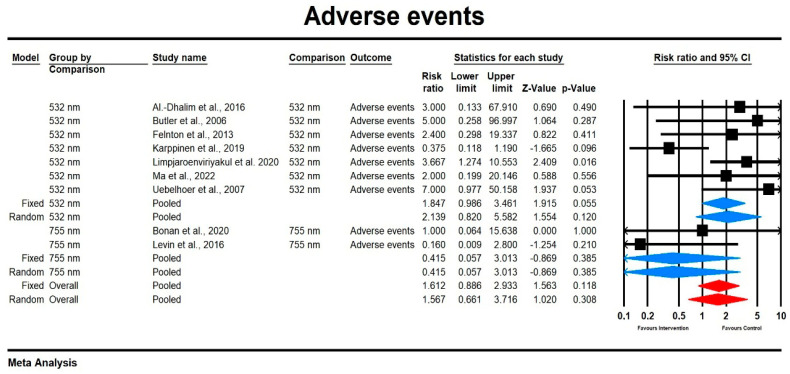
The effect size (RR) for the adverse events in patients subjected to 532/755 nm (intervention) vs. comparator (control) [15,17,18,19,20,22,23,25,29].

Using a mixed-effects model, we found that the relative risk for adverse events was moderate but did not significantly differ across laser types (RR = 1.567, 95% CI [0.661, 3.716], *p* = 0.383). The heterogeneity among studies was low, with I^2^ = 31.298% and a non-significant Q-value (Q = 14.084, *p* = 0.118). Subgroup analysis showed that the effect size was higher for the 532 nm laser compared to the 755 nm laser, although neither was statistically significant (755 nm: RR = 0.415, 95% CI [0.057, 3.013], *p* = 0.385; 532 nm: RR = 2.139 95% CI [0.820, 5.582], *p* = 0.12). The results are displayed in Figure 5. The sensitivity analysis results suggest that individual studies do not substantially impact the overall effect. Funnel plot inspection revealed no significant publication bias, as confirmed by Egger’s test (*p* = 0.602), with a symmetric plot indicating minimal bias (see Supplemental Figure S4).

#### 3.3.4. Patient Satisfaction

Patient satisfaction with laser treatments varied by laser type, modality, and treatment area. Across studies, patient satisfaction generally mirrored the efficacy presented above.

In Al-Dhalimi and Al-Janabi’s study (2016) [15], satisfaction was assessed via patient self-reports, showing high satisfaction levels with the 532 nm Nd: YAG laser over the 1064 nm laser for port wine stain treatment (7.6 vs. 3.3. in a patient 10-point satisfaction score collected via interview). Bohnert et al. (2018) [16] reported higher satisfaction in the arm treated with the 532 nm laser in their study on solar lentigines (82.5% vs. 75%). In Limpjaroenviriyakul et al.’s study [20], a higher satisfaction measured as a changed score was found for the 532 nm device whilst in and Ma et al. [22], higher study scores were noted for the comparative laser (mean score of 8.11) over the 532 nm picosecond Nd: YAG laser (mean score of 6.72). However, in the study by Nam et al. [21], a patient self-assessment using a six-point satisfaction scale produced results which were comparable.

Levin et al. (2016) [29] found 84% of patients were “satisfied” to “completely satisfied” with Q-switched lasers compared to 50% for the picosecond alexandrite, using a 7-point Likert scale, while Uebelhoer et al. (2007) [23] showed greater satisfaction with the 532 nm in terms of the overall effect, downtime, and experience. Bonan et al. (2021) [25] measured satisfaction on a 5-point Likert scale and demonstrated that patients reported greater satisfaction with MoveoPL, scoring it higher (average score of 3.6 for both treatments). However, 93% of the patients reported a preference for MoveoPL, whereas only 7% preferred the standard SP treatment. In the study by Lee et al. (2012) [28], 44% of patients reported “excellent” or “good” satisfaction with the 755 nm long-pulsed alexandrite laser in treating pigmentary lesions, assessed via self-assessment questionnaires. In Lee et al.’s paper (2018) [27], satisfaction for melasma treatment was higher for the 755 nm alexandrite laser than the Q-switched Nd: YAG laser.

##### Patient Satisfaction Score and Relative Risk

In two studies using the 532 nm laser [15,22], patient satisfaction scores were a reported endpoint, with a pooled SMD of 0.785; SE = 1.692; 95% CI: [−2.532, 4.102]. However, the effect size did not differ significantly by laser type (*p* = 0.643). The heterogeneity between the studies was high, with I^2^ = 96.716% and a significant Q-value (Q = 30.449, *p* = 0.000).

For satisfaction as a qualitative measure, two other studies [16,19] provided data, with a pooled RR of 1.233; 95% CI: [0.814, 1.867]; Z = 0.989. The heterogeneity among these studies was low, with I^2^ = 0.0% and a non-significant Q-value (Q = 0.249, *p* = 0.617).

### 3.4. Meta-Regression Analyses

For endpoint data (SMD) regarding 532 nm laser efficacy, *p* values for pulse duration (Q = 3.18, df = 2, *p* = 0.204) and type of lesion (Q = 2.02, df = 2, *p* = 0.3635) were non-significant, indicating that the predicted effect sizes were not dependent on any of these variables. Compared to millisecond pulse duration, the picosecond pulse duration showed a negative non-significant association with treatment efficacy (coefficient = −1.0263; Z = −1.22; *p* = 0.22), while the nanosecond pulse duration showed a positive but non-significant association (coefficient = 0.9351; Z = 1.00; *p* = 0.3195). In the case of lesion type, the coefficients were positive and as follows: pigmented: −1.36, Z = −1.40, *p* = 0.1601; vascular: −0.5887, Z = −0.74, *p* = 0.4606. When we conducted meta-regression for RR of improvement, the *p* values were also non-significant for vascular vs. pigmented lesions (Q = −1.872, Z = −1.28, *p* = 0.2022).

For the adverse event outcome related to the 532 nm laser (risk ratio), pulse duration was not a significant moderator (Q = 0.01, df = 2, *p* = 0.9955). However, the overall model examining lesion type was statistically significant (Q = 9.46, df = 2, *p* = 0.0088), indicating that lesion type significantly moderates the treatment effect. Specifically, when comparing vascular to pigmented lesions, the coefficient for vascular lesions was −1.8687 (*p* = 0.0085), suggesting a significantly lower effect size—i.e., a reduced risk of adverse events—for vascular lesions. The model explained approx. 71% of the heterogeneity. No additional meta-regression analyses were feasible based on the available data in the present meta-analysis.

### 3.5. Risk of Bias

Table 3 assesses the risk of bias in each study across seven domains, highlighting key trends, which are as follows:

1. **Random Sequence Generation (Selection Bias):** All studies demonstrated a “Low Risk” of selection bias, suggesting that the randomization process was robust.
2. **Allocation Concealment (Selection Bias):** Most studies maintained a low risk by ensuring allocation concealment, though a few studies were rated as “Unclear” due to insufficient reporting.
3. **Blinding of Participants and Personnel (Performance Bias):** While blinding was achieved in several studies, approximately one-third had a “High Risk” due to lack of blinding of participants and personnel, which could potentially impact performance bias.
4. **Blinding of Outcome Assessment (Detection Bias):** Most studies ensured low risk for outcome assessment blinding, although some had “Unclear” or “High Risk” ratings due to partial or no blinding of the outcome assessors.
5. **Incomplete Outcome Data (Attrition Bias):** Nearly all studies were rated as “Low Risk”, indicating good data completeness. Only one study had unclear attrition data, which may affect short-term outcomes.
6. **Selective Reporting (Reporting Bias):** All studies showed a low risk of reporting bias, suggesting consistency in reporting all specified outcomes.
7. **Other Bias:** Only one study presented a potential source of other bias due to a device manufacturer affiliation, while all others were free from additional noted biases.

The ROB analysis thus suggests that the studies generally adhered to good research practices, particularly in randomization, allocation concealment, and data completeness. Some areas, notably participant and personnel blinding, could be improved for stronger bias mitigation across all domains.

## 4. Discussion

This meta-analysis, comprising 16 studies with 509 participants involved, comparatively evaluated the efficacy and safety of 532 nm and 755 nm lasers in treating pigmented and vascular lesions on the face. This meta-analysis demonstrated that overall improvement scores did not significantly differ by laser type (SMD = 0.014, SE = 0.25, 95% CI [−0.476, 0.504], *p* = 0.906); however, high heterogeneity was present (I^2^ = 86.7%). Subgroup analysis, however, indicated a notable efficacy advantage for the 755 nm laser (532 nm: SMD = −0.027, SE = 0.429, 95% CI [−0.867, 0.813], *p* = 0.895; 755 nm: SMD = 0.035, SE = 0.308, 95% CI [−0.569, 0.639], *p* = 0.909), with a significant relative improvement (755 nm: RR = 1.512, 95% CI [1.070, 2.136], *p* = 0.019). The observation hereby confirms the greater efficacy of treating pigmentary and vascular lesions on the face with a 755 nm device. Our group previously demonstrated an optimal clearance of melasma-type pigmented lesions and mild hyperpigmented skin lesions with a 755 nm laser, but with a simultaneous reduction in the temperature effect, by using a very short pulse duration in the picosecond range [31]. From our perspective, additionally, 1064 nm lasers with a pulse duration shortened to picoseconds can be effective. A short picosecond pulse duration compared to the widely used millisecond pulse duration is very important in the treatment of pigmented lesions because it minimizes the risk of inflammation associated with temperature PIH (post-inflammatory hyperpigmentation) [32]. However, in the treatment of vascular lesions, it is necessary to achieve an optimal temperature that effectively coagulates and closes the vessel, so it is reasonable to use a pulse duration in the millisecond range [33]. Overall, it seems that the more pronounced efficacy of the 755 nm laser results from effective melanin-targeting, deeper penetration, and reduced thermal damage, making it more versatile for treating both pigmented and vascular lesions compared to the more superficial 532 nm laser.

Pain scores between the two lasers were found to be significantly different in this meta-analysis (SMD = −1.104, SE = 0.543, 95% CI [−2.168, 0.064], *p* = 0.042) with high heterogeneity (I^2^ = 95.977%). It turned out that the 532 nm device produces more a painful experience (532 nm: SMD = −1.336, SE = 0.636, 95% CI [−2.582, −0.09], *p* = 0.036; 755 nm: SMD = −0.480, SE = 1.043, 95% CI [−2.524, 1.564], *p* = 0.645). Specific studies echoed these findings, as reviewed in the Results section. This variability in pain perception underscores the importance of considering patient comfort and sensitivity to different wavelengths when determining treatment protocols. Unfortunately, the use of anesthetic cream was not consistently reported across all included studies. Some studies explicitly stated that no anesthetic was applied, while others did not provide this detail. Where available, we included such information in the data extraction table. However, due to inconsistent reporting, we could not control for this variable in the meta-analysis—not enough studies provided data with regard to pain scores to be able to conduct subgroup analyses/meta-regression. Other safety outcomes across studies were generally favourable, with minimal adverse events reported. No significant difference in the relative risk of adverse events was observed between the lasers (RR = 1.567, 95% CI [0.661, 3.716], *p* = 0.383), and heterogeneity was moderate (I^2^ = 31.289%). However, some studies noted slightly higher incidences of transient adverse events, such as erythema and blistering, with the 532 nm laser, which may be attributable to its higher absorption rate in superficial tissues, as confirmed with subgroup analysis (755 nm: RR = 0.415, 95% CI [0.057, 3.013], *p* = 0.385; 532 nm: RR = 2.139, 95% CI [0.820, 5.582], *p* = 0.12). In most studies, the authors suggested that while both lasers maintain acceptable safety profiles, selecting the appropriate wavelength based on the lesion’s depth and patient skin type can optimize safety and efficacy [34,35].

The choice between 532 nm and 755 nm lasers for treating facial pigmented and vascular lesions should be guided by specific clinical considerations, as each wavelength interacts differently with skin chromophores and tissue depths [16,25]. As confirmed earlier, the 532 nm laser is absorbed more by superficial chromophores. Thus, it is primarily effective for epidermal pigmented lesions and superficial vascular conditions but may not reach deeper targets. However, shorter wavelength, higher absorption, and thus greater energy deposition in the epidermis—which contains more melanin and a higher density of nerve endings—can lead to more scattered heat, increasing the risk of surrounding tissue damage and, consequently, pain. This higher absorption rate by melanin can increase the risk of post-inflammatory hyperpigmentation, especially in individuals with darker skin types where melanin content is naturally elevated, making the 532 nm laser more challenging to use in these populations without causing pigmentation changes [22]. Also, typically, the 532 nm laser is used for smaller spot sizes and thus more energy cumulates in the skin as compared to larger 755 nm laser spots [35,36,37,38,39]. Indeed, the meta-regression analysis also demonstrated that 532 nm laser treatment of vascular lesions was associated with significantly fewer adverse events compared to pigmented lesions (coefficient = −1.8687, *p* = 0.0085). This suggests that vascular lesion treatments using 532 nm lasers may be better tolerated overall. A plausible explanation lies in the underlying tissue characteristics and laser–tissue interaction mechanisms as described above. Vascular lesions primarily involve superficial dermal blood vessels, where hemoglobin serves as the primary chromophore. Hemoglobin absorbs 532 nm light efficiently, allowing for effective treatment at relatively low energy settings and with shorter pulse durations, thereby minimizing collateral thermal damage to surrounding tissues [35]. Conversely, the 755 nm wavelength penetrates deeper into the skin and is less absorbed by melanin, which makes it especially suitable for treating deeper pigmented lesions, such as melasma, and for patients with darker skin types [26,28]. This wavelength bypasses superficial melanin to minimize the risk of hyperpigmentation. Consequently, this provides a safer option for those with higher melanin levels who are at increased risk of pigmentary side effects when treated with shorter wavelengths [21,24]. Studies have demonstrated the efficacy of the 755 nm wavelength in managing various benign hyperpigmentations, with evidence of high patient satisfaction and favorable outcomes, such as higher visual analog scores (VAS) and greater clearance in melasma cases [25,28].

While it is indeed clinically intuitive and already known that factors such as pulse duration and other laser parameters, as well as the type of lesion treated influence treatment outcomes and represent important sources of variability, we performed meta-regression analysis to evaluate whether pulse duration (ps vs. ns vs. ms) and lesion type (pigmented, vascular, or mixed) may significantly influence treatment efficacy and contribute to variability across studies. These findings highlight that variations in laser parameters beyond wavelength—particularly pulse duration—are clinically relevant and should be carefully considered in both research design and clinical practice. For efficacy at 532 nm, although we observed a trend toward smaller effect sizes with picosecond lasers and in the treatment of vascular lesions, this did not reach statistical significance, likely due to the limited number of available studies. Additionally, for the outcome of adverse events at 532 nm, the treatment of vascular lesions was significantly associated (*p* = 0.02) with a reduced effect size, indicating a lower risk of experiencing adverse events as described above. Overall, accounting for these moderators helps to explain some of the heterogeneity observed in treatment outcomes and reinforces the importance of individualized parameter selection based on lesion characteristics and not only wavelength but other laser parameters. Consequently, clinicians should carefully consider lesion type, lesion depth, skin type, and patient preferences when selecting the appropriate laser wavelength for treatment to maximize efficacy while minimizing adverse effects, as well as pain. Selecting a laser that aligns with the depth and chromophore concentration of the lesion can optimize treatment outcomes and reduce post-treatment complications, especially in diverse skin types where pigmentation risks differ [16,23,26]. Additionally, adjusting laser parameters, such as energy settings and pulse duration, may further tailor treatment to the unique needs of the patient, enhancing safety and satisfaction in laser-based dermatologic procedures [21,30]. It is believed that laser setting parameters such as energy density and pulse duration are fundamental, but they should be expanded to include treatment spot size, which affects the depth of light penetration in the skin, and pulse emission frequency, which affects temperature, i.e., the risk of PIH and pain.

Challenges in data extraction across the studies complicated this analysis, largely due to inconsistencies in outcome reporting. In several studies, data were either missing or presented differently between text and figures, making it challenging to ensure data reliability and comparability. Nam et al. (2019) [21], for example, displayed outcome measurements inconsistently across sections of the study, and subjective measures such as patient satisfaction and improvement ratings varied widely across studies due to differences in scale and assessment methodology. In the study by Butler et al. (2006) [17], patients rated IPL higher for pigment lesions despite KTP’s better performance for vascular lesions, demonstrating how subjective reporting variability impacts outcome comparability. This lack of uniformity underscores the importance of standardized outcome measures in future research to improve reliability and comparability in laser therapy studies.

The limitations of this meta-analysis, including high heterogeneity, small sample sizes, and variability in subjective outcome measures, should be acknowledged. For instance, Fabi et al. (2014) [26] reported wide variability in MMASI scores for melasma, reflecting subjective scoring, while Felton et al. (2014) [18] observed lesion recurrence in some patients after 10 months, underscoring the importance of long-term follow-up data. Moreover, regarding pain severity assessment, inconsistent reporting prevented us from accounting for the use of anesthetic cream prior to the procedure, as too few studies provided such data to allow for subgroup analyses or meta-regression. Lastly, at the screening phase, some of the studies might have been missed. Another important limitation of this meta-analysis is the variability and, in some cases, the lack of long-term follow-up data across the included studies. As the durability of treatment effects is particularly relevant in the management of pigmented lesions—where recurrence can occur over time—this limits our ability to draw conclusions regarding the sustained efficacy of 532 nm and 755 nm lasers. Due to inconsistent reporting and the variability of observation periods following the laser treatment cycle in the majority of trials, we were unable to perform subgroup analyses based on follow-up duration. We therefore recommend that future studies incorporate standardized and sufficiently long follow-up periods to better evaluate the long-term outcomes of laser therapies, especially in the context of pigmented lesion treatment. Finally, while no significant publication bias was detected, the limited number of studies restricts generalizability.

Future research should focus on larger, standardized samples with consistent methodologies and explore long-term outcomes, such as recurrence and quality of life, to strengthen the evidence for clinical applications of 532 nm and 755 nm lasers in facial lesion treatments. Future studies in hyperpigmented lesions should be expanded to include a clear division of efficacy depending on pulse duration in the millisecond, nanosecond, and picosecond ranges, and additionally correlated with the appropriate laser wavelength. Regarding vascular lesions, only pulse duration in the millisecond range seems effective, but an objective comparison of the efficacy of individual laser wavelengths with the broadband effect of pulsed light devices, so-called IPL, seems reasonable. Importantly, we recommend that future trials standardize and clearly report the use of anesthetics, particularly when pain is assessed as a primary outcome. Additionally, they should focus on larger, standardized trials with consistent methodologies and examine long-term outcomes such as disease recurrence and quality of life to strengthen the evidence for the clinical use of 532 nm and 755 nm lasers in the treatment of facial lesions.

In conclusion, this meta-analysis concludes that both 532 nm and 755 nm lasers are effective in treating facial pigmented and vascular lesions, with the 755 nm laser showing a slight advantage and 532 nm producing more painful experiences but less adverse events in the case of vascular lesions. We believe that our work contributes novel insights by providing a structured, evidence-based foundation for optimizing laser therapy and supports the call for individualized treatment based on both clinical and technical factors.

## Figures and Tables

**Table 1:** Study characteristics.

Study Characteristics			Intervention							Comparator							Sample Characteristics			
Publication Year/Country	Sponsorship/Blinding/ Study Type	N Total Randomized/Analyzed	Laser 1 Name	Length of Wave [nm]	Fluence [J/cm^2^]	Spot Size [mm]	Frequency [Hz]	Pulse Duration [ps]	Anesthetic Cream Applied (Y/N)	Laser 2 Name	Length of Wave [nm]	Fluence [J/cm^2^]	Spot Size [mm]	Frequency [Hz]	Pulse Duration [ps}	Intervention Duration	Age: Mean/SD	N Males	Type of Lesion	Fitzpatrick Type (List Type with n)
Al-Dhalim et al., 2016/Iraq [15]	A/None/SF	19/14	Nd: YAG	532	30	3	1	25 [ms]	Y	Nd: YAG	1064	300	3	2	20 [ms]	6 every 4 weeks	22.07/9.003	1	Facial port wine stain	III-3, IV-11
Bohnert et al., 2018/USA [16]	A/DB/SF	10/10	KTP/Nd: YAG	532/1064	4; 4.5	3; 6	10	ND	Y	Nd: YAG	1064	4	6	10	ND	6 every 2–3 weeks	55/(40–63) range	0	Mild-to-severe facial mottled pigmentation	I-1, II-7, III-2
Butler et al., 2006/USA [17]	Ind/DB/SF	19/19	KTP	532	7–10	10	ND	15–30 [ms]	N	IPL (Starlux)	IPL	25–36	10 × 15	ND	20 [ms]	ND	Over 18 years old/ND	ND	Pigmented and/or vascular dyschromias	ND
Felton et al., 2014/England [18]	A/None/Other participants	21/15 (11 for 532 and 755)	Nd: YAG-532 nm	532	0.5–5	2–5	ND	50 [ns]	Y	QS alexandrite (Alex)—755 nm	755	4.2–18	2–4	ND	100 [ns]	Median 8 (4–13)	24 (median)/15–61 (range)	5	Nevus of Ota (NO)	II-2, IV-5, V-8
Karppinen et al., 2019/Multicenter [19]	A/DB/SF	24/18	KTP	532	20–30	1	ND	10 [ms]	N	Yellow laser—PhotoLase (585 nm)	585	5.6–8.1	1.4	ND	25 [ms]	1–2 every 1–2 months	48/27–62 (range)	2	Telangiectasia	I-6, II-8, III-4
Limpjaroenviriyakul et al., 2020/England [20]	Ind/SB/Other participants	30/30	Q-switched Nd: YAG 532 nm laser (QS 532-nm)	532	1	2	3	ND	Y	Low-fluence Q-switched Nd: YAG 1064 nm laser (LFQS 1064 nm)	1064	2.4	6	5	ND	1064 nm: 5 every 2 weeks, 532 nm: once	27.73/7.91	8	Idiopathic hyperpigmented lip	ND
Nam et al., 2019/Korea [21]	A/DB/SF	20/19	Fractional 532 nm KTP laser	532	3.86	20 × 20 [mm^2^]	ND	5 [ms]	ND	595 nm pulsed dye laser (PDL)	595	7.5	10	ND	6 [ms]	3 every 4 weeks	41.5/21–59 (range)	5	Facial erythema and telangiectasia	ND
Ma et al., 2022/China [22]	A/DB/SF	18/18	QSAL-treated group or picosecond 532 nm Nd: YAG	532	0.5–0.6	3	2 [MHz]	375	Y	Q-switched alexandrite laser	755	5–6	3	2 [MHz]	100 ± 10 [ns]	1	27.11/4.81	3	Freckles	III-14, IV-4
Uebelhoer et al., 2007/USA [23]	Ind/SB/SF	15/14	532 nm KTP laser (Gemini, Laserscope)	532	10; 9	5; 10	ND	23 [ms]	ND	595 nm flashlamp-pumped, long-pulsed PDL (V-Beam, Candela)	595	7.5	10	1	10 [ms]	3 every 3 weeks	52.4/35–70 (range)	8	Facial telangiectasias, diffuse telangiectatic facial erythema	I-6, II-6, III-2, IV-1
West and Alster 1998/USA [24]	A/None/Other participants	20/17	KTP (532 nm) laser (Aura; Laserscope, San Jose, CA, USA)	532	15	1	ND	10 [ms]	ND	590 or 595 nm long-pulse dye laser (ScleroPlus Laser; Candela Corporation, Wayland, MA, USA)	590, 595	15; 20	2 × 7, 2 × 7	ND	1.5 [ms]	1 or 2 (8-week interval)	40/23–69	0	Facial or leg telangiectasia	I-Nd, II-Nd, III-Nd
Bonan et al., 2021/Italy [25]	Ind/SB/SF	63/63	MoveoPL 755 alexandrite laser	755	18–25	ND	2.5–3.5	ND	ND	Standard SP emission	SP	18–25	5–10	1–1.5	ND	2 every 50 days	57/12	9	Benign hyperpigmentation, pigmentation scars	I-16, II-12, III-19, IV-16
Fabi et al., 2014/USA [26]	Ind/DB/SF	20/16	Low-fluence QSAL	755	2.0, 1.8, 1.2, 1.1	8	5	ND	ND	Low-fluence Q-switched Nd: YAG	1064	2.0	8	5	ND	6 every 1 week	43.4/32–64 (range)	1	Melasma	II-2, III-7, IV-11
Lee et al., 2018/England [27]	Ind/None/SF	12/12	755 nm picosecond alexandrite laser	755	0.88–1.18	4.4–5.1	ND	650	ND	1064 nm QS-Nd: YAG	1064	2.0, 3.5, 3.2	8, 6, 4	ND	ND	4 every 1 month	ND/32–52 (range)	1	Melasma	III-Nd, IV-Nd
Lee et al., 2012/Korea [28]	A/None/Two groups based on type of lesions on the face ((i) those with pigmentation; and (ii) those with facial flushing, skin laxity or some pigmentation.)	116/116	Long-pulsed 755 nm alexandrite	755	125	1.5–18	ND	0.25–0.5 [ms]	Y	Long-pulsed 1064 nm Nd: YAG	1064	40–50	10	ND	0.25–300 [ms]	1	40.73/2.35	6	Facial flushing and telangiectasia	III-Nd, IV-Nd
Levin et al., 2016/USA [29]	Ind/TB/Side-by-side	42/42	755 nm alexandrite picosecond laser	755	0.71–4.07	2.5–6	ND	750–900	ND	Q-switched frequency-doubled 532 nm Nd: YAG nanosecond, Q-switched 694 nm ruby nanosecond	532, 694, 1064	ND	ND	ND	ND	picosecond: 4.12; nanosecond: 5.46 treatments	37.1/1–71 (range)	6	Nevus of Ota	III-16, IV-11, V-14, VI-1
Zhang et al., 2023/China [30]	A/SB/SF	86/86	Picosecond alexandrite laser (PicoSure; Cynosure)	755	3.77–4.80	2.3–2.6	1	550–750	ND	Ti:sapphire laser (PicoWay; Syneron-Candela)	730	3.75	2-3	1	250	1	30.7/21–62 (range)	7	Freckles	III-16, IV-70

A—academia; DB—double-blind; SF—split face; ND—no data; Ind—industry; SB—single-blind.

**Table 2:** Improvement scores in meta-analyzed studies.

Improvement/Clinical Efficacy Endpoint							
Publication Year	Name of Tool	Laser 1 Ø	Laser 1 ±	Laser 1 n	Laser 2 Ø	Laser 2 ±	Laser 2 n
532 nm							
Al-Dhalim et al., 2016 [15]	VAS 5-point visual analog scale assessment for halves of PWS with photos	2.28	1.43	14	3.71	0.82	14
Bohnert et al., 2018 [16]	Global Aesthetic Improvement scale (1–5) with CR VISIA	1.35	0.37	19	0.76	0.37	10
Felton et al., 2014 [18]	ND	90	6.32	5	74.28	28.7	10
Nam et al., 2019 [21]	Colorimetric Endpoint Analysis: CR-400 device (Minolta, Tokyo, Japan)	12.77	2.77	19	12.22	2.75	19
Nam et al., 2019 [21]	Clinical photography endpoint analysis: 10-point Percent Change Scale	2.89	1.71	19	2.79	1.13	19
Ma et al., 2022 [22]	Photographic Endpoint Analysis: Five-grade Percent Change Scale	3.11	0.583	18	3.78	0.428	18
Uebelhoer et al., 2007 [23]	Photographic Endpoint Analysis: Five-grade Percent Change Scale	85	nd	8	75	nd	8
West and Alster 1998 [24]	Photographic Evaluation: assessed by a physician and nurse.	1.45	nd	20	3	nd	20
755 nm							
Bonan et al., 2020 [25]	Grading score (0–4) using LifeViz digital imaging system	3.6	0.6	63	2.8	0.8	63
Lee et al., 2018 [27]	VAS-5-point visual analog scale assessment	1.38	0.48	12	2.04	0.75	12
Levin et al., 2016 [29]	VAS 5-point visual analog scale assessment	2.44	nd	8	2.57	nd	22
Zhang et al., 2023 [30]	Photographic Evaluation via VISIA-CR camera (Canfield Scientific)	69.27	7.75	86	68.99	7.42	86
Zhang et al., 2023 [30]	Global Aesthetic Improvement Scale (GAIS) score (5-point scale)	4.02	0.3	86	4.04	0.31	86
Felton et al., 2014 [18]	Endpoint Analysis of Improvement: percentage-based evaluation	75	18.02	6	82.22	29.73	9

Laser 1—intervention laser; Laser 2—control device.

**Table 3:** The risk of bias in the studies in the present meta-analysis.

Author	Random Sequence Generation (Selection Bias)	Allocation Concealment (Selection Bias)	Blinding of Participants and Personnel (Performance Bias)	Blinding of Outcome Assessment (Detection Bias) (Mortality)	Incomplete Outcome Data Addressed (Attrition Bias) (Short-Term Outcomes)	Selective Reporting (Reporting Bias)	Other Bias
Al-Dhalimi and Al-Janabi (2016) [15]	Low Risk—Randomization by simple draw	Low Risk—Half-lesion treatment	High Risk—Not blinded	High Risk—Outcome assessors not blinded	Unclear—Attrition not fully explained (5 dropouts)	Low Risk	None noted
Bohnert et al. (2018) [16]	Low Risk—Randomized split-face study	Low Risk—Side randomization	Low Risk—Double-blinded	Low Risk—Blinded investigator assessments	Low Risk—All participants completed	Low Risk	None noted
Bonan et al. (2021) [25]	Low Risk—Randomization conducted	Unclear Risk—Allocation details unclear	Low Risk—Controlled and blinded	Low Risk—Outcome assessments were blinded	Low Risk—High completion rate	Low Risk	None noted
Butler et al. (2006) [17]	Low Risk—Randomized, split-face design	Low Risk—Clear allocation	Low Risk—Blinding maintained	Low Risk—Blinded assessors	Low Risk—All participants completed	Low Risk	None noted
Fabi et al. (2014) [26]	Low Risk—Randomization applied	Low Risk—Allocation well managed	Low Risk—Double-blind protocol	Low Risk—Independent evaluations	Low Risk—High follow-up rate for each group	Low Risk	None noted
Felton et al. (2014) [18]	Low Risk—Randomization via pre-test patches	Low Risk—Allocated by laser response	Unclear Risk—No personnel blinding details	Low Risk—Independent evaluations	Low Risk—High follow-up completion	Low Risk	None noted
Karppinen et al. (2019) [19]	Low Risk—Randomized, double-blinded	Low Risk—Blinded side allocation	Low Risk—Double-blinded design	Low Risk—Blinded assessors	Low Risk—All participants completed	Low Risk	Device bias (manufacturer affiliation)
Limpjaroenviriyakul et al. (2020) [20]	Low Risk—Randomized controlled study	Low Risk—Allocation by block	High Risk—No blinding reported	Unclear Risk—No specific blinding for outcome assessors	Low Risk—All patients completed study	Low Risk	None noted
Zhang et al. (2023) [30]	Low Risk—Randomized, split-face controlled	Low Risk—Controlled side allocation	Low Risk—Blinded split-face trial	Low Risk—Blinded outcome assessments	Low Risk—Complete dataset	Low Risk	None noted
Lee et al. (2012) [28]	Low Risk—Randomized split treatment	Unclear Risk—Allocation not specified	High Risk—No blinding	Low Risk—Blinded assessors	Low Risk—Full data presented	Low Risk	None noted
Lee et al. (2018) [27]	Low Risk—Randomized, split-face design	Unclear Risk—Random allocation unclear	High Risk—Blinding not reported	Unclear-No blinding for outcome assessment	Low Risk—High retention rate	Low Risk	None noted
Levin et al. (2016) [29]	Low Risk—Retrospective analysis	Unclear Risk—Allocation not described	High Risk—No blinding for personnel	Low Risk—Blinded evaluation of outcomes	Low Risk—Complete data presented	Low Risk	Retrospective design
Ma et al. (2022) [22]	Low Risk—Randomized split-face study	Low Risk—Clear allocation	High Risk—No blinding	Low Risk—Blinded outcome assessors	Low Risk—All participants completed	Low Risk	None noted
Nam et al. (2019) [21]	Low Risk—Randomized controlled study	Unclear Risk—Allocation methods unclear	High Risk—No blinding for personnel	Low Risk—Outcome assessment blinded	Low Risk—High completion rate	Low Risk	None noted
Uebelhoer et al. (2007) [23]	Low Risk—Randomized, split-face design	Low Risk—Clear allocation	Low Risk—Single-blinded for participants	Low Risk—Blinded outcome assessments	Low Risk—High retention rate	Low Risk	None noted
West and Alster (1998) [24]	Low Risk—Randomized, split-face comparison	Low Risk—Clear allocation	Low Risk—Single-blinding for patients	Low Risk—Independent blinded evaluation	Low Risk—All data complete	Low Risk	None noted

## Data Availability

No new data were created in this manuscript.

## Supplementary Materials

The following supporting information can be downloaded at: https://www.mdpi.com/article/10.3390/jcm14082546/s1, Figure S1: Funnel Plot of Standard Error by Std Diff in Means, Figure S2: Funnel Plot of Standard Error by Log Risk Ratio, Figure S3: Funnel Plot of Standard Error by Std Diff in Means, Figure S4: Funnel Plot of Standard Error by Log Risk Ratio.

## Abbreviations

The following abbreviations are used in this manuscript:

CI	Confidence Interval
Cochran Risk-of-Bias Tool	Cochrane Risk-of-Bias Tool for Randomized Trials
CINAHL	Cumulative Index to Nursing and Allied Health Literature
Embase	Excerpta Medica Database
IPL	Intense Pulsed Light
I^2^	I-Squared Statistic
KTP laser	Potassium Titanyl Phosphate Laser
MMASI	Melasma Area and Severity Index
Nd: YAG laser	Neodymium-Doped Yttrium Aluminum Garnet Laser
Nd laser	Neodymium Laser
OC	Observed Cases
PIH	Post-inflammatory Hyperpigmentation
PDL	Pulsed Dye Laser
PL	Photolysis
PRISMA	Preferred Reporting Items for Systematic Reviews and Meta-Analyses
Q Statistic, Q-value	Q Statistic
Q-switched laser	Q-switched Laser
RCTs	Randomized Controlled Trials
RR	Risk Ratio
ROB	Risk of Bias
SE	Standard Error
SMD	Standardized Mean Difference
SP	Standard Pulsed
τ^2^	Between-Study Variance
USA	United States of America
VAS	Visual Analog Scale
WHO	World Health Organization
n	Number
nm	Nanometer
*p*	*p*-value
MoveoPL mode	Moveo Photo Light Mode
PubMed	Public/Publisher MEDLINE
