# Supplementary figures and images for: The Efficacy of 532/755 nm Laser Therapy for Facial Pigmented and Vascular Lesions: A Systematic Review and Meta-Analysis
Source: J Clin Med. 2025 Apr 8;14(8):2546. doi: 10.3390/jcm14082546 (PMC12028165; doi:10.3390/jcm14082546)

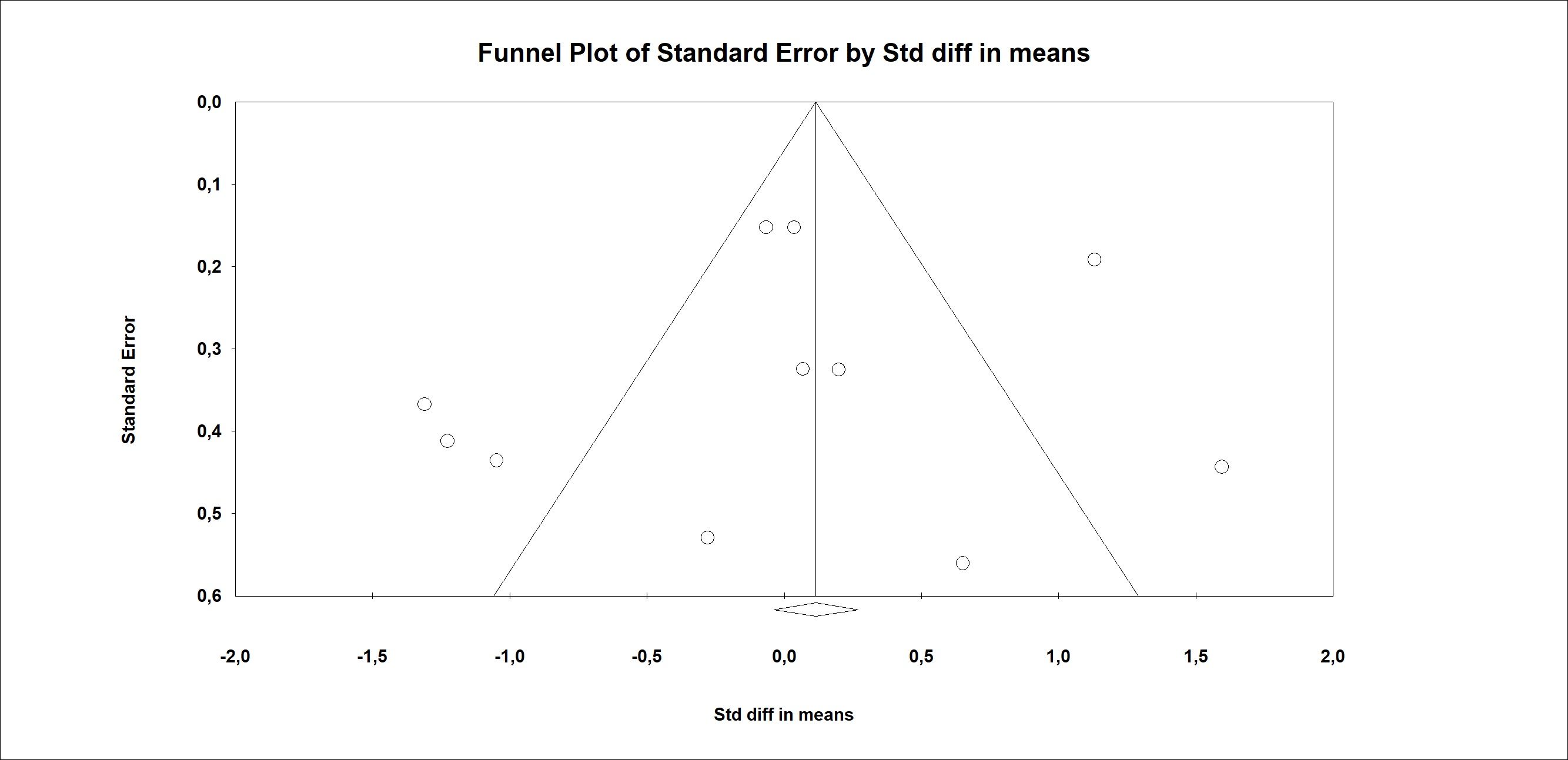

Supplement: Supplementary file 1 [file jcm-14-02546-s001.zip › suppl figure 1.jpg]

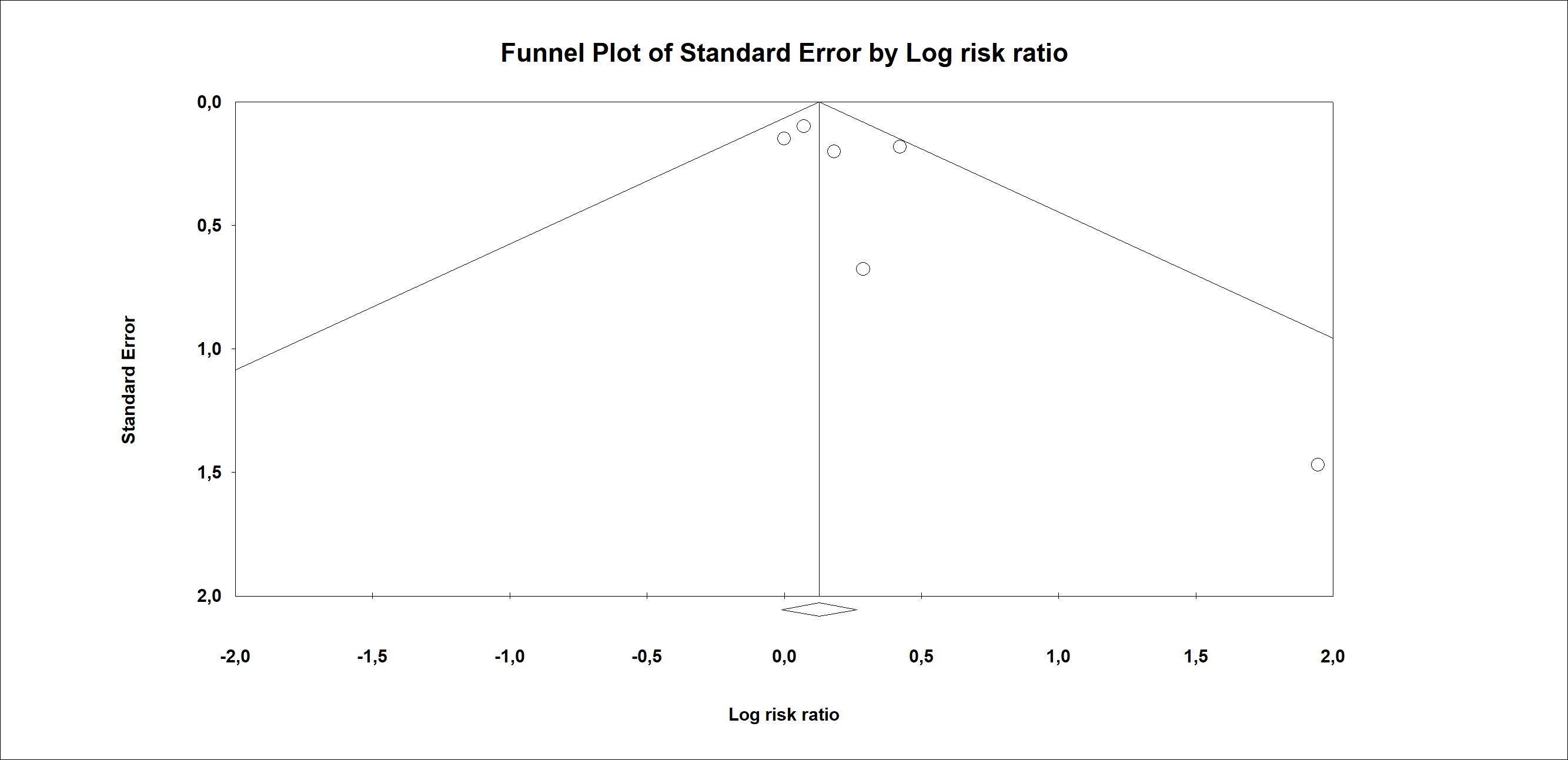

Supplement: Supplementary file 1 [file jcm-14-02546-s001.zip › suppl figure 2.jpg]

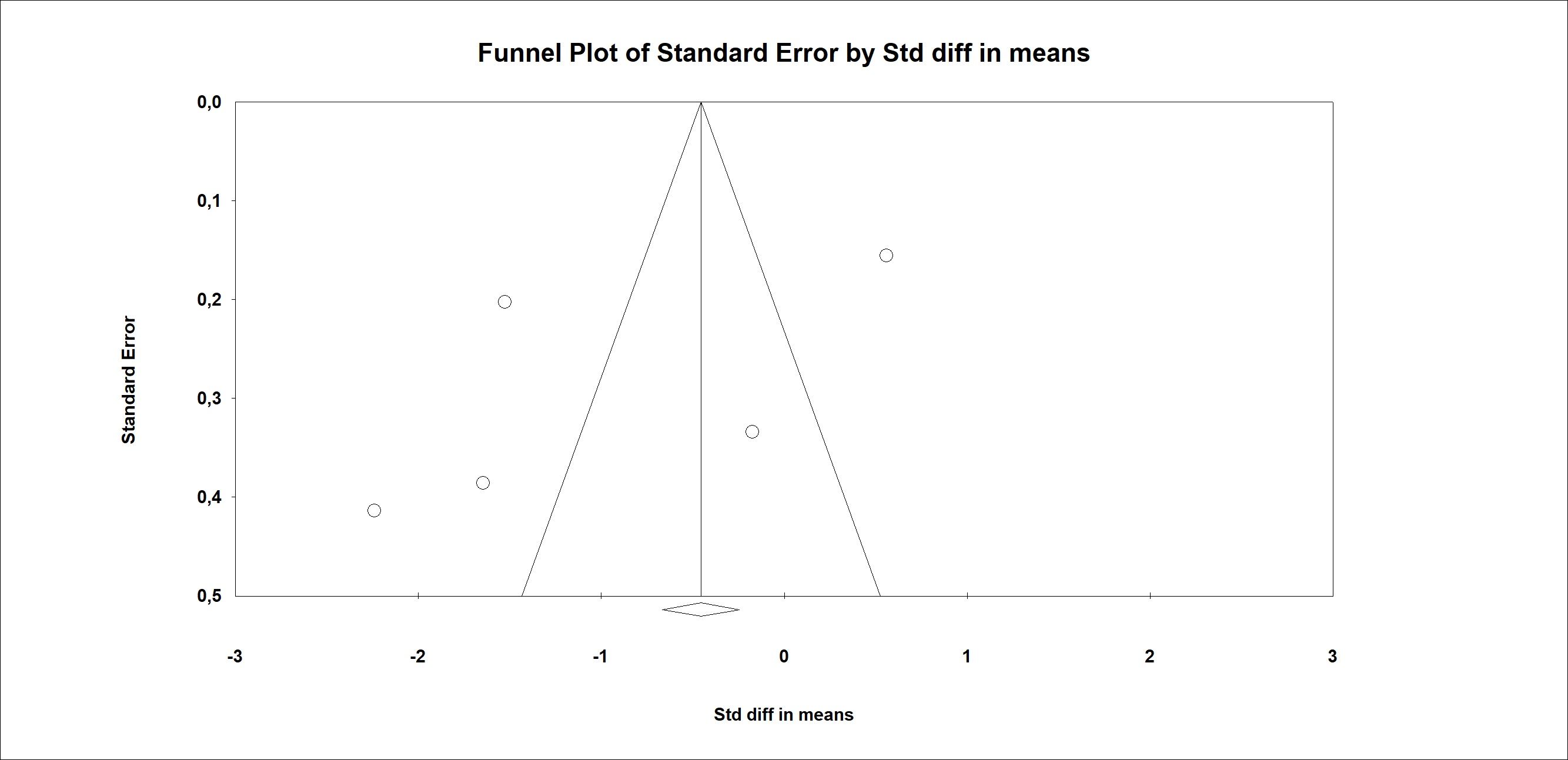

Supplement: Supplementary file 1 [file jcm-14-02546-s001.zip › suppl figure 3.jpg]

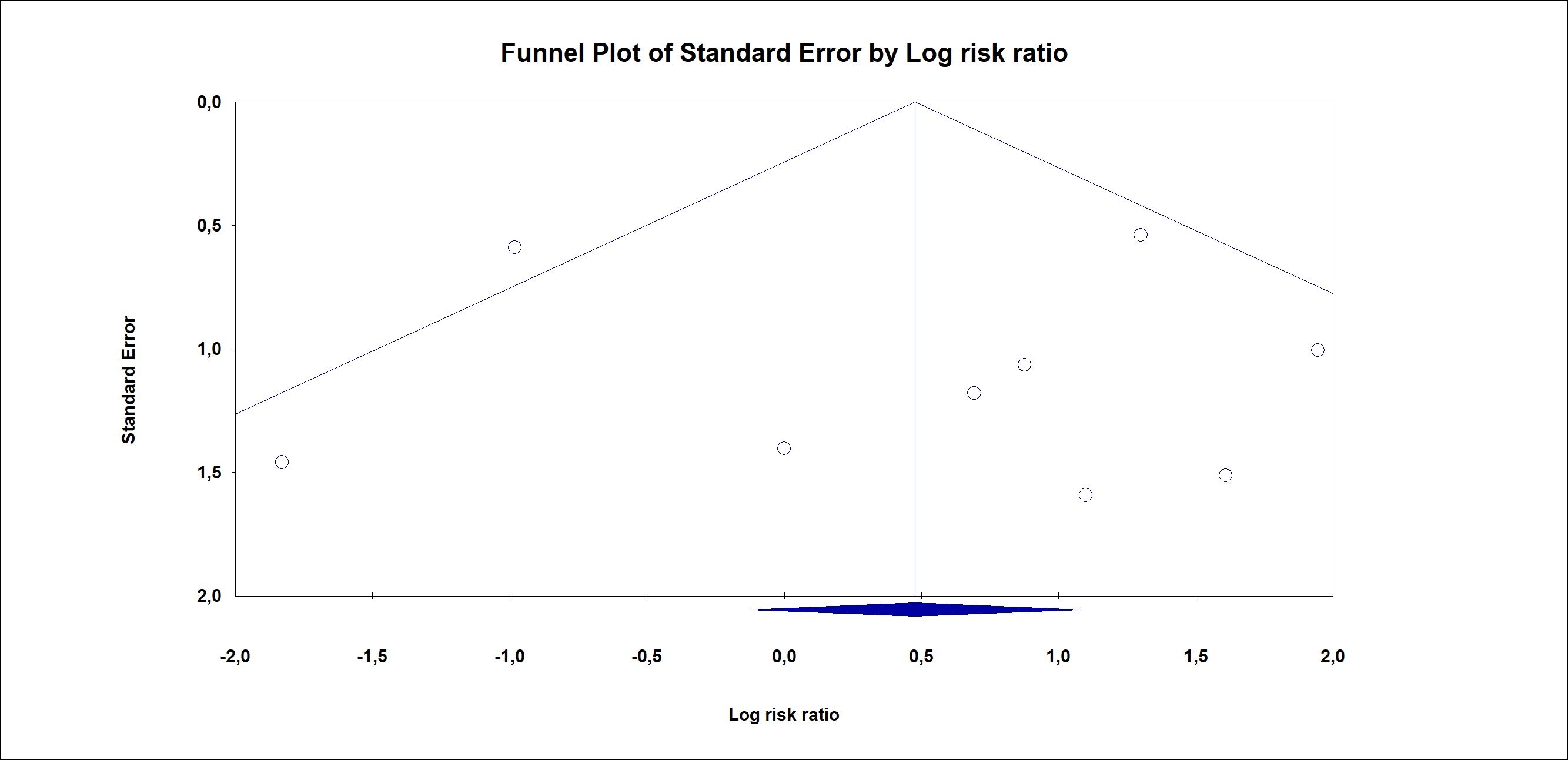

Supplement: Supplementary file 1 [file jcm-14-02546-s001.zip › suppl figure 4.jpg]
